# Supplementary material for: Pyruvate Dehydrogenase Kinase 1 inhibition mediated oxidative phosphorylation enhancement in cartilage promotes osteoarthritis progression
Source: BMC Musculoskelet Disord. 2023 Jul 20;24:597. doi: 10.1186/s12891-023-06585-6 (PMC10357736; doi:10.1186/s12891-023-06585-6)
Supplement: Supplementary file 1 — Additional file 1. [file 12891_2023_6585_MOESM1_ESM.zip › BP.Gsea.1653623667859/GOBP_QUINONE_METABOLIC_PROCESS.html]

Details for gene set GOBP\_QUINONE\_METABOLIC\_PROCESS[GSEA]

|  || Dataset | 55457\_collapsed\_to\_symbols.cls.cls #OA\_versus\_normal.cls.cls #OA\_versus\_normal\_repos |
| Phenotype | cls.cls#OA\_versus\_normal\_repos |
| Upregulated in class | OA |
| GeneSet | GOBP\_QUINONE\_METABOLIC\_PROCESS |
| Enrichment Score (ES) | 0.3968853 |
| Normalized Enrichment Score (NES) | 1.4200063 |
| Nominal p-value | 0.05459057 |
| FDR q-value | 0.4720952 |
| FWER p-Value | 1.0 |
Table: GSEA Results Summary

  

Fig 1: Enrichment plot: GOBP\_QUINONE\_METABOLIC\_PROCESS      
 Profile of the Running ES Score & Positions of GeneSet Members on the Rank Ordered List

  

| SYMBOL | TITLE | RANK IN GENE LIST | RANK METRIC SCORE | RUNNING ES | CORE ENRICHMENT || 1 | COQ9 | coenzyme Q9 [Source:HGNC Symbol;Acc:HGNC:25302] | 561 | 0.636 | 0.0194 | Yes |
| 2 | CBR1 | carbonyl reductase 1 [Source:HGNC Symbol;Acc:HGNC:1548] | 598 | 0.624 | 0.0783 | Yes |
| 3 | AKR1A1 | aldo-keto reductase family 1 member A1 [Source:HGNC Symbol;Acc:HGNC:380] | 808 | 0.567 | 0.1182 | Yes |
| 4 | CRYZL1 | crystallin zeta like 1 [Source:HGNC Symbol;Acc:HGNC:2420] | 847 | 0.555 | 0.1702 | Yes |
| 5 | NDUFA9 | NADH:ubiquinone oxidoreductase subunit A9 [Source:HGNC Symbol;Acc:HGNC:7693] | 943 | 0.529 | 0.2151 | Yes |
| 6 | FDXR | ferredoxin reductase [Source:HGNC Symbol;Acc:HGNC:3642] | 1536 | 0.423 | 0.2110 | Yes |
| 7 | NQO2 | N-ribosyldihydronicotinamide:quinone reductase 2 [Source:HGNC Symbol;Acc:HGNC:7856] | 1568 | 0.418 | 0.2499 | Yes |
| 8 | COQ4 | coenzyme Q4 [Source:HGNC Symbol;Acc:HGNC:19693] | 1824 | 0.381 | 0.2679 | Yes |
| 9 | COQ2 | "coenzyme Q2, polyprenyltransferase [Source:HGNC Symbol;Acc:HGNC:25223]" | 1884 | 0.375 | 0.3003 | Yes |
| 10 | AKR1C3 | aldo-keto reductase family 1 member C3 [Source:HGNC Symbol;Acc:HGNC:386] | 1996 | 0.361 | 0.3275 | Yes |
| 11 | COQ3 | "coenzyme Q3, methyltransferase [Source:HGNC Symbol;Acc:HGNC:18175]" | 2164 | 0.342 | 0.3483 | Yes |
| 12 | VKORC1 | vitamin K epoxide reductase complex subunit 1 [Source:HGNC Symbol;Acc:HGNC:23663] | 2306 | 0.326 | 0.3697 | Yes |
| 13 | CBR4 | carbonyl reductase 4 [Source:HGNC Symbol;Acc:HGNC:25891] | 2565 | 0.295 | 0.3788 | Yes |
| 14 | AKR7A2 | aldo-keto reductase family 7 member A2 [Source:HGNC Symbol;Acc:HGNC:389] | 2690 | 0.280 | 0.3969 | Yes |
| 15 | AKR1B1 | aldo-keto reductase family 1 member B [Source:HGNC Symbol;Acc:HGNC:381] | 3138 | 0.236 | 0.3856 | No |
| 16 | PDSS2 | decaprenyl diphosphate synthase subunit 2 [Source:HGNC Symbol;Acc:HGNC:23041] | 3453 | 0.207 | 0.3816 | No |
| 17 | COQ8A | coenzyme Q8A [Source:HGNC Symbol;Acc:HGNC:16812] | 4359 | 0.124 | 0.3235 | No |
| 18 | CYP4F11 | cytochrome P450 family 4 subfamily F member 11 [Source:HGNC Symbol;Acc:HGNC:13265] | 5144 | 0.060 | 0.2686 | No |
| 19 | AKR1C4 | aldo-keto reductase family 1 member C4 [Source:HGNC Symbol;Acc:HGNC:387] | 5843 | 0.005 | 0.2149 | No |
| 20 | COQ6 | "coenzyme Q6, monooxygenase [Source:HGNC Symbol;Acc:HGNC:20233]" | 7156 | -0.092 | 0.1221 | No |
| 21 | AKR1C1 | aldo-keto reductase family 1 member C1 [Source:HGNC Symbol;Acc:HGNC:384] | 8198 | -0.171 | 0.0581 | No |
| 22 | AKR1B10 | aldo-keto reductase family 1 member B10 [Source:HGNC Symbol;Acc:HGNC:382] | 8420 | -0.187 | 0.0594 | No |
| 23 | COQ7 | "coenzyme Q7, hydroxylase [Source:HGNC Symbol;Acc:HGNC:2244]" | 9169 | -0.245 | 0.0256 | No |
| 24 | CYP4F3 | cytochrome P450 family 4 subfamily F member 3 [Source:HGNC Symbol;Acc:HGNC:2646] | 9224 | -0.250 | 0.0462 | No |
| 25 | CYP4F2 | cytochrome P450 family 4 subfamily F member 2 [Source:HGNC Symbol;Acc:HGNC:2645] | 9599 | -0.283 | 0.0451 | No |
| 26 | PDSS1 | decaprenyl diphosphate synthase subunit 1 [Source:HGNC Symbol;Acc:HGNC:17759] | 9750 | -0.296 | 0.0628 | No |
| 27 | COQ10B | coenzyme Q10B [Source:HGNC Symbol;Acc:HGNC:25819] | 10099 | -0.331 | 0.0685 | No |
| 28 | UBIAD1 | UbiA prenyltransferase domain containing 1 [Source:HGNC Symbol;Acc:HGNC:30791] | 11299 | -0.471 | 0.0219 | No |
| 29 | CYP4F12 | cytochrome P450 family 4 subfamily F member 12 [Source:HGNC Symbol;Acc:HGNC:18857] | 11423 | -0.490 | 0.0609 | No |
| 30 | COQ8B | coenzyme Q8B [Source:HGNC Symbol;Acc:HGNC:19041] | 11763 | -0.544 | 0.0883 | No |
Table: GSEA details [plain text format]

  

Fig 2: GOBP\_QUINONE\_METABOLIC\_PROCESS      
 Blue-Pink O' Gram in the Space of the Analyzed GeneSet

  

Fig 3: GOBP\_QUINONE\_METABOLIC\_PROCESS: Random ES distribution      
 Gene set null distribution of ES for **GOBP\_QUINONE\_METABOLIC\_PROCESS**
